# Quorum Quenching Activity of the PGPR *Bacillus subtilis* UD1022 Alters Nodulation Efficiency of *Sinorhizobium meliloti* on *Medicago truncatula*

**DOI:** 10.3389/fmicb.2020.596299

**Published:** 2021-01-15

**Authors:** Amanda Rosier, Pascale B. Beauregard, Harsh P. Bais

**Affiliations:** ^1^Department of Plant and Soil Sciences, University of Delaware, Newark, DE, United States; ^2^Delaware Biotechnology Institute, University of Delaware, Newark, DE, United States; ^3^Département de Biologie, Université de Sherbrooke, Sherbrooke, QC, Canada

**Keywords:** PGPR, symbiosis, consortia, legume, quorum sensing, quorum quenching, nodule, agriculture

## Abstract

Plant growth-promoting rhizobacteria (PGPR) have enormous potential for solving some of the myriad challenges facing our global agricultural system. Intense research efforts are rapidly moving the field forward and illuminating the wide diversity of bacteria and their plant beneficial activities. In the development of better crop solutions using these PGPR, producers are including multiple different species of PGPR in their formulations in a “consortia” approach. While the intention is to emulate more natural rhizomicrobiome systems, the aspect of bacterial interactions has not been properly regarded. By using a tri-trophic model of *Medicago truncatula* A17 Jemalong, its nitrogen (N)-fixing symbiont *Sinorhizobium meliloti* Rm8530, and the PGPR *Bacillus subtilis* UD1022, we demonstrate indirect influences between the bacteria affecting their plant growth-promoting activities. Co-cultures of UD1022 with Rm8530 significantly reduced Rm8530 biofilm formation and downregulated quorum sensing (QS) genes responsible for symbiotically active biofilm production. This work also identifies the presence and activity of a quorum quenching lactonase in UD1022 and proposes this as the mechanism for non-synergistic activity of this model “consortium.” These interspecies interactions may be common in the rhizosphere and are critical to understand as we seek to develop new sustainable solutions in agriculture.

## Introduction

Legume crops are an essential component of sustainable agriculture due to their multifaceted benefits to ecology and human health (Stagnari et al., 2017). This is attributable to the mutualism between symbiotic N-fixing bacteria (*Rhizobia*) and their specific legume plant hosts, referred to as “biological nitrogen fixation” (BNF). *Rhizobia* fix atmospheric N in exchange for carbon-rich photosynthates within specialized structures formed on the plant root called nodules (Oldroyd, 2013). Peoples et al. (2009) estimates that 30–40 kg of N is fixed per ton of crop legume dry matter and Herridge et al. (2008) approximates that N-fixation by crop and forage legumes via symbiosis globally is roughly 50 Tg per year. The ability of *Rhizobia* to fix nitrogen within an agricultural setting is a key factor in de-coupling dependence on synthetic nitrogen application. However, for BNF to effectively replace N-fertilization, a clear understanding of the numerous mechanisms increasing BNF efficiency is required.

The interspecies signaling pathway between legumes and their bacterial symbionts responsible for BNF is well described, especially in the model legume *Medicago truncatula*, which is closely related to the forage crop alfalfa. Symbiosis between *M. truncatula* and *Sinorhizobium meliloti* commences through root exudation of the signaling plant flavonoid luteolin, which acts as a chemoattractant (Hassan and Mathesius, 2012). Luteolin induces transcription and expression of *S. meliloti nod* genes, producing lipo-chitooligosaccharide signals termed Nod factors (NFs) (Kondorosi et al., 1989). Receptors localized at the root hair recognize these NFs, instigating bacterial invasion and nodule organogenesis (Oldroyd et al., 2011; Gourion et al., 2015; Zipfel and Oldroyd, 2017); *S. meliloti* then divide, proliferate, and express N_2_-fixing nitrogenase enzyme within the plant-derived nodules (Oldroyd, 2013). Legume symbiosis clearly relies on a finely tuned system of molecular pathways between bacteria and host.

For example, a key factor for the successful initiation of nodulation is the production of exopolysaccharides (EPS) by *S. meliloti* (Marketon et al., 2003). EPS include succinoglycan as well as high- and low-molecular-weight molecules of galactoglucan (EPS II) present in *S. meliloti* biofilms (Rinaudi and Gonzalez, 2009). The EPS II fraction within these biofilms are described as “symbiotically active”; EPS II defective mutants are unable to form pink nodules (González et al., 1996). EPS II production is dependent on the quorum sensing (QS) regulatory network including *expR* (Pellock et al., 2002) and *sinI* genes (Marketon et al., 2003). The *S. meliloti* ExpR/SinI QS system relies on SinI synthase-produced long-chain N-acyl homoserine lactone (AHL) signal molecules (Marketon et al., 2002; Gao et al., 2005). The AHL-bound ExpR protein controls the expression of ∼500 QS genes (Gurich and González, 2009). One of these genes of note, *wggR*, encodes a transcriptional regulator activating the downstream *wge* operons responsible for the biosynthesis and polymerization of EPS II low-molecular-weight galactoglucans, the symbiotically active EPS component of *S. meliloti* biofilm (Gao et al., 2012). This QS pathway is a highly controlled intraspecies mode of communication that is crucial for *S. meliloti* to successfully coordinate activities at a community level. Considering these and the other myriad molecular communications occurring in the rhizosphere, it is imperative to inquire how other bacteria may be influencing these core symbiotic pathways.

Indeed, millions of different species of bacteria inhabit the ecosystem on and around plant roots, termed the “rhizosphere” (Adesemoye et al., 2009; Gamalero and Glick, 2011). Organisms within the rhizosphere improving plant health and resilience directly or indirectly are known as plant growth-promoting rhizobacteria (PGPR) (Kloepper et al., 1980; Pii et al., 2015). Many bacteria have been identified as generalist PGPRs for their association with a broad range of plants, and their specific plant beneficial activities have been described. *Bacillus* species are highly researched generalist PGPRs known to promote plant growth through their ability to solubilize nutrients and produce phytohormones, antifungal secondary metabolites, and volatile organic compounds (VOCs) (Aloo et al., 2019). Several *Bacillus* species are also known to express quorum quenching (QQ) enzymes, disrupting QS signaling of other bacteria, including pathogens (Dong et al., 2001; d’Angelo-Picard et al., 2005; Ryan et al., 2009). These activities are promising areas of rhizomicrobiome research as they may have unexpected influences on complex interspecies interactions.

Due to their numerous modes of action, PGPRs such as *Bacillus* have been utilized for crop applications and are a growing proportion of agrochemical company research efforts, where they are broadly termed “biologicals” (Timmusk et al., 2017; Marrone, 2019). Biological global markets are expanding (Arora et al., 2020), and the potential of these products is driving new market formulations incorporating multiple different species of live bacteria in “consortia” (Marrone, 2019). The rationale behind this innovation stems from the knowledge that natural rhizomicrobiomes are occupied by millions of different species of bacteria working in conjunction with one another (Schlatter et al., 2015); restoration of these microbial ecosystems may provide more robust benefits to the plant (Gouda et al., 2018; Sergaki et al., 2018). This concept, described as “synergism”, manifests as “additive” plant benefits observed when multiple PGPRs are applied as compared to a single PGPR.

Synergistic plant growth promotion by multiple PGPR species has been observed in certain plant–bacteria–bacteria interspecies systems (Schwartz et al., 2013; Morel et al., 2015; Berendsen et al., 2018) but has also failed to produce in others (Felici et al., 2008; Kang et al., 2014; Maymon et al., 2015). Various co-inoculation combinations of *Bacillus* species and *Rhizobia* on legumes have shown synergistic growth and nodulation outcomes. Most prominently, nodulation was significantly enhanced in soybean (Glycine max L. Merr) systems when *Bradyrhizobium japonicum* was co-inoculated with *B. cereus* UW85 (Halverson and Handelsman, 1991), *B. thuringiensis* NEB17 (Bai et al., 2003), or *B. amyloliquefaciens* strain LL2012 (Masciarelli et al., 2014). *B. subtilis*-specific co-inoculations have been successful when used with *Rhizobium leguminosarum* bv. *viciae* 128C53 (Rlv) onto *Pisum sativum* L. (pea) (Schwartz et al., 2013) and when applied along with *B. japonicum* on soybean (Bai et al., 2003). No direct mechanisms of interaction were queried in these studies. Identifying suitable PGPR consortia requires understanding the multitude of plant beneficial activities that may be altered when the organisms coexist in what is now more commonly being described as the plant holobiont (Zilber-Rosenberg and Rosenberg, 2008). The lack of identified PGPR interspecies interaction mechanisms remains a significant gap in our knowledge, yet poses an opportunity to pursue empirical selections of appropriate PGPRs as we continue to expand our understanding of their plant beneficial activities.

To investigate meaningful legume–PGPR mechanisms, we designed a simplified tri-trophic legume–symbiont–PGPR system consisting *of M. truncatula* A17 Jemalong, its symbiotic mutualist *S. meliloti* strain Rm8530, and the PGPR *B. subtilis* strain UD1022 (Glazebrook and Walker, 1989; Bishnoi et al., 2015; Rosier, 2016). The organisms in this model were specifically selected to be representative due to their comprehensively described genetics and lifestyles. The primary goal of this work is to identify interspecies interactions between PGPR, which may influence their plant beneficial activities in the legume–*Rhizobia* symbiosis. By using the tri-trophic model as a platform for testing phenotypic outcomes of the “consortium,” more fundamental questions regarding the interspecies interactions can be developed. Specifically, does the PGPR and legume symbiont consortia act synergistically to increase *M. truncatula* plant growth, how do the different PGPR species directly or indirectly interact with one another, and do those interactions influence their ability to interact with and confer benefits to the plant? We employed phenotypic and molecular assays to evaluate the legume–*Rhizobia*–PGPR interactions.

## Materials and Methods

### Bacterial Growth

Primary cultures of all bacteria strains were grown and maintained on TYC media [TY media (Beringer, 1974) liquid or agar supplemented with 1 mM CaCl_2_] with appropriate antibiotics. Subcultures of *S. meliloti* strain Rm8530 and *Bacillus subtilis* strain UD1022 (hereafter “UD1022”) prepared for biofilm treatments were sub-cultured into minimal glutamate mannitol (MGM) and low phosphate (0.1 mM), as described in Marketon and Gonzalez (2002). Both UD1022 and Rm8530 strains were grown at 30°C for all experiments. AT medium for culturing pre-induced *A. tumefaciens* KYC55 was prepared as described in Joelsson and Zhu (2005). Strains used in this work are listed in Table 1.

**TABLE 1:** Bacterial strains used in this study.

Strain	Genotype	References or Sources
Rm1021	SU47 *str-21 expR102::ISRm2011-1*	Meade et al., 1982
Rm8530	*Sinorhizobium meliloti* Rm1021 *expR*^+^	Glazebrook and Walker, 1989
Rm8530 SinI-gfp	with integrated pMG309	Gao et al., 2012
Rm8530 WggR-gfp	with integrated pMG310	Gao et al., 2012
KYC55	*Agrobacterium tumefaciens*	Zhu et al., 2003
	(pJZ410) (pJZ384) (pJZ372)	
UD1022	*Bacillus subtilis*	Bishnoi et al., 2015
UD1022 *ytnP*^–^	UD1022*ytnP*::erm	Dr. Pascale Beauregard

### Plant Growth and Co-inoculation

Seeds of *M. truncatula* A17 cv Jemalong were acid scarified for 6 min and sterilized with 3% bleach for 3 min. Seeds were imbibed in sterile water at 4°C overnight, rinsed and placed in sterile petri dish, and germinated covered overnight at room temperature (Garcia et al., 2006). Germinated seeds were placed in sterile Magenta^®^ (Magenta Corp.) jars with Lullien’s solution (Lullien et al., 1987), sealed with 3M^TM^ MicroPore^TM^ surgical tape, and grown in a controlled environmental chamber at 55% relative humidity and a 14 h, 22°C day/10 h, 18°C night cycle. After 6 days of growth, plants were inoculated with bacteria treatments, with 10 plants per treatment. Rm8530 was grown to OD_600_ = 0.8 and UD1022 was grown at OD_600_ = 1.0. Bacteria were spun down, washed three times in sterile H_2_O, and resuspended with 0.5 × Lullein’s solution with Rm8530 final OD_600_ = of 0.02 and UD1022 OD_600_ = 0.01 (in Magenta jar). Plants were harvested 7 weeks after inoculation. Experiment was repeated three times.

### Cross-Streak for Growth Inhibition Analysis

Rm8530 bacteria were grown to OD_600_ = 0.8 and UD1022 OD_600_ = 1.5. Both cultures were diluted to OD_600_ = 0.5 with sterile H_2_O. Bacteria were streaked on TYC agar plates using a sterile loop in a cross pattern.

### Biofilm Assays

#### Preparation of Cell-Free Supernatant (CFS) Derived From UD1022 for Biofilm Assays

UD1022 was inoculated from a single plate colony into 5 ml of TYC and grown overnight (16 h) and then diluted 1:50 in 50 ml of MGM in a sterile 150 ml flask and grown shaking for 8 h to an OD_600_ = 0.8–1.0. Cultures were centrifuged 10 min, 4°C at 4,000 RPM. Culture supernatant was filter-sterilized with 0.22 μm membrane (Steriflip^®^, EMD Millipore) under gentle vacuum. Supernatant was centrifuged and filter-sterilized once more. A sub-fraction was heat treated in water bath overnight at 65°C.

#### Preparation of Biofilm Treatments

Biofilm assays were based on methods found in O’Toole et al. (1999) and Rinaudi and Gonzalez (2009). Rm8530 was grown 48 h in TYC to OD_600_ = 1.5–2.0, and then cells were “pre-conditioned” by diluting 1:100 to MGM media and grown shaking 48 h to OD_600_ = 0.8. Stocks of treatments were made by centrifuging and re-suspending cell pellets with fresh MGM, or UD1022 CFS, UD1022 “heat-treated” CFS to a total of 5% by volume in MGM. One hundred microliters of these treatment stocks was then aliquoted to 96-well plates with eight replicate wells per treatment. Plates were sealed with Parafilm^®^ (Bemis Company, Inc.) and placed in a shaker at 30°C and measured at 24, 48, and 72 h. Experiment was repeated three separate times.

Plates were then emptied and gently rinsed three times with sterile water, dried, and stained 20 min with 150 μl of 0.1% crystal violet. Plates were emptied, rinsed gently three times with sterile water. Crystal violet (CV) was solubilized with modified biofilm dissolving solution (MBDS) (Tram et al., 2013). OD_570_ of CV was then measured using Wallac 1420 Plate Reader (PerkinElmer Life and Analytical Science, Wallac Oy, P.O. Box 10, FIN-20101 Tuku, Finland).

### Gene Expression Reporter Assays

Reporter lines for Rm8530 were provided by Dr. Max Teplitski of the University of Florida. All cultures grown in liquid TYC broth shaking at 225 RPM at 30°C. Bacteria primary cultures were grown with appropriate antibiotics 48 h to OD_600_ = 2.0–3.0. Cells were further prepared as described in the *Biofilm Assays* section and 29 replicate wells were included per treatment. Every 24 h, total well fluorescence and cell growth were measured using Wallac 1420 Plate Reader (PerkinElmer Life and Analytical Science, Wallac Oy, P.O. Box 10, FIN-20101 Tuku, Finland). Data were reported as fluorescence counts/OD_570_ (Gao et al., 2012). After the 72 h measurement, 96-well plates were processed as described in the *Biofilm Assays* section above to assess qualitative biofilm formation. Gene reporter assays were repeated three times.

### Statistical Analysis

For plant growth biological data and biofilm analysis, data normality and homogeneity were reviewed prior to analysis of variance (ANOVA). No data transformations were required. One-way ANOVA was used to test for differences between treatments. When *F* ratios were significant (*p* < 0.05), treatment means were compared via Tukey Kramer HSD using SAS-JMP (Cary, NC, United States).

For gene expression reporter results analysis, ANOVA was used to test for treatment differences. Where *F*-ratios were significant (*p* < 0.05), treatment means were compared via Tukey–Kramer test (JMP, SAS Institute Inc, 1989-2019). Non-parametric analyses (Kruskal–Wallis test) were utilized if data failed to meet parametric assumptions. Where *H*-values (Kruskal–Wallis test statistic) were significant (*p* < 0.05), treatment means were compared via Kruskal–Wallis multiple comparison *Z*-value test using NCSS software (Hintze, 2000).

### Gene Expression Analysis Using Semi-Quantitative Reverse Transcription PCR (qRT-PCR)

#### Primer Design for qRT-PCR

Gene sequences were derived from GenBank; *S. meliloti* 1021 sequences were derived from genome (accession: AL591688.1) and mega-plasmids pSymA (accession: AE006469.1). The *sinI* primer pair from Gurich and González (2009) and the *rpo*E1 primer pair from Trabelsi et al. (2009). Primers from this work were designed using GenScript Real-time PCR (TaqMan) Primer Design^1^. Amplicon size was restricted to 150 bp or less. All primer sequences (Table 2) were cross-checked on all strain sequences to ensure species specificity.

**TABLE 2:** Primer sequences used in this study.

Primer	Sequence 5′-3′	Amplicon	Source length
Rm8530 rpoE1-fw	CGAGGAAGAGGTCCTGGAAT	100 bp	Trabelsi et al., 2009
Rm8530 rpoE1-rv	GACGCAGTCCTGCAACAGAT		
Rm8530 SinI F	CCGGAAATCCGTAGTGCGTC	76 bp	Gurich and González, 2009
Rm8530 SinI R	ATGCGCGATCCTGGGAGATT		
Rm8530 WggR F	TCCGTTCGCAGACTTTGGAG	107 bp	This work
Rm8530 WggR R	CGAGCGAATCATCTCCGTCA		

#### Experimental Protocol for qRT-PCR

For qRT-PCR analysis, cells were “pre-conditioned” on MGM media as described under the *Biofilm Assays* section. Cells were pelleted and re-suspended in fresh MGM plus the treatment. Co-inoculations were combined as Rm8530 OD_600_ = 0.8 and UD1022 OD_600_ = 0.2. Luteolin treatments contained a final concentration of 5 μM luteolin. Treatments were grown shaking at 30°C, and 1.5 ml samples were collected at time points of 12 and 24 h, centrifuged, decanted, and flash frozen in liquid nitrogen. RNA was isolated using NucleoSpin^®^ RNA from Macherey-Nagel (Düren, Germany). cDNA was generated with 500 ng of RNA using High Capacity cDNA Reverse Transcription Kit from Applied Biosystems^2^ and qPCR was performed using PerfeCTa^®^ SYBR^®^ Green SuperMix, ROX, Quanta Biosciences (Gaithersburg, MD), and run on Eppendorf Mastercycler^®3^ ep *realplex*^2^. Experiments were repeated three times.

#### Expression Analysis of qRT-PCR

The relative change in gene expression was calculated with the 2^–ΔΔ*Ct*^ method as described in Schmittgen and Livak (2008), which calculates the expression of the gene of interest relative to the internal control in the treated sample compared with the untreated control. The internal control gene for Rm8530 is *rpo*E1. Genes were considered to be differentially expressed if the fold change in expression was ≥2 or ≤ -2.

### AHL Biosensor Assays for QQ Analysis

Preparation of the AHL biosensor *Agrobacterium tumefaciens* KYC55 was as described in Joelsson and Zhu (2005) with modifications. KYC55 pre-induced cells were inoculated 1:1,000 into MGM medium for X-Gal soft agar 6-well plates. Pre-induced KYC55 cells were made as described in Joelsson and Zhu (2005). Soft agar plates were treated the same day they were poured. UD1022 was inoculated from a fresh plate streaked from glycerol stock into TYC and grown shaking 30°C for 5 h to OD_600_ = 1.5, then sub-cultured 1:100 to MGM media and grown shaking 30°C for 20 h to OD_600_ = 0.5. Treatments were made using these cultures mixed into sterile micro-centrifuge tubes with standard C8-AHL and 3-oxo-C16-AHL to a final concentration of 10 μM in a volume of 200 μl. Controls contained standard AHL only. Treatments were incubated shaking 30°C for 24 h. Samples were then centrifuged at 16,000 × *g* for 10 min at 4°C. Supernatants were transferred to new sterile tubes and sterilized open in a biosafety cabinet under UV light for 30 min. Two microliters of treatments was applied to KYC55 X-Gal soft agar six-well plates and allowed to dry. Two treatment replicates were included on two separate six-well plates. AHL biosensor assay was repeated twice.

### Sequence Homology and Alignment

The FASTA protein sequence YtnP protein in *B. subtilis* subsp. *subtilis* str. 168 (sequence NP_390867.1) was queried using tblastn search translated nucleotide databases using a protein query for *B. subtilis* UD1022 nucleotide reference sequence (NZ_CP011534.1). The protein sequence for UD10222 YtnP has 281 amino acids and has a molecular weight of 31.8 kDa. The alignment of UD1022 YtnP, AiiA, and other MBL sequences was performed in MEGA (Tamura et al., 2011) by using the software MUSCLE (Edgar, 2004).

### Construction of YtnP Mutant

The *ytnP* gene disruption *B. subtilis* subsp. *subtilis* trpC2 *ytnP::erm* (Koo et al., 2017) was obtained from the Bacillus Genetic Stock Center and transferred into *B. subtilis* UD1022 by SPP1 phage transduction (Yasbin and Young, 1974).

### YtnP Protein Expression and Purification

The *B. subtilis* UD1022 *ytnP* specific sequence was submitted to University of North Carolina School of Medicine Center for Structural Biology (NIH grant P30CA016086) for protein expression and purification. Workers sent the sequence to GenScript for gene synthesis and subcloning into a pET expression vector that contains an N-terminal His tag followed by a TEV site for tag removal during purification [pET-28a(+)-TEV]. The *ytnP*::*E. coli* construct expression was done at UNC using their autoinduction expression system. Purification was performed using a Ni-affinity step, TEV protease tag removal, subtractive Ni-affinity step to separate out the tag, and size-exclusion chromatography to remove potential protein contaminants.

## Results

### Co-inoculation of UD1022 and Rm8530 Do Not Synergistically Promote Plant Growth

**FIGURE 1:**
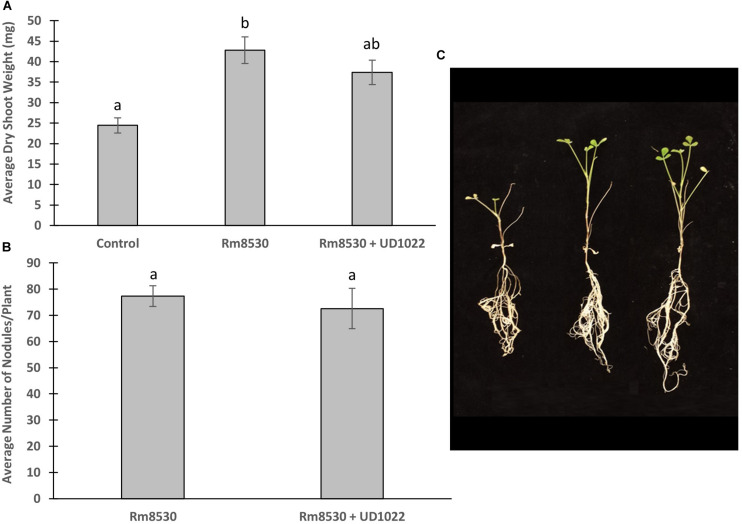
Co-inoculated plant growth and nodulation. **(A)** Average plant dry weight of Rm8530 treated control plants and co-inoculated Rm8530 & UD1022 plants did not differ statistically (*p*-value of 0.06). **(B)** There was no statistical difference between average counts of nodules between treatments (*p*-value of 0.59). **(C)** Overall plant growth of both treatments was greater than control (first plant), but no differences were observed between Rm8530 treatment (second plant) and Rm8530 & UD1022 co-inoculation (third plant).

*M. truncatula* plants were co-inoculated with *B. subtilis* UD1022 and *S. meliloti* Rm8530 6 days after germination and analyzed 7 weeks after inoculation for biomass and nodulation. Though the co-inoculation of Rm8530 and UD1022 resulted in no statistical difference in shoot biomass (Figure 1A, *p* = 0.06), there was a slight decrease in observable shoot growth (Figure 1C). There was no statistical difference in nodule numbers (Figure 1B, *p* = 0.59) between co-inoculated plants from those inoculated with Rm8530 alone. These results indicate that the addition of the PGPR UD1022 to the symbiotic strain Rm8530 did not increase plant health, contrary to results from similar studies (Fox et al., 2011; Morel et al., 2015). We speculated that the lack of growth promotion by the co-inoculation may be due to the antagonistic activity of UD1022 against Rm8530. A standard cross-streak compatibility assay on solid media determined no direct growth inhibitory effect between the two bacteria (Supplementary Figure S1).

### UD1022 Interacts Indirectly With Rm8530 by Interfering With Rm8530 Biofilm and QS

**FIGURE 2:**
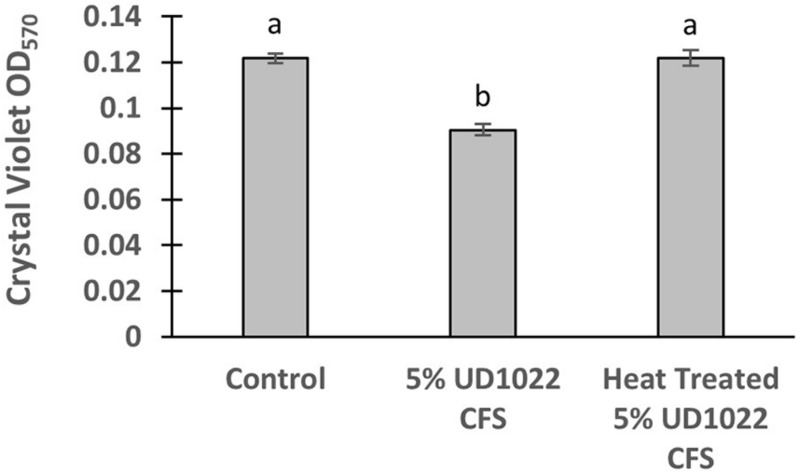
Rm8530 biofilm formation assay. Treatment with 5% UD1022 CFS significantly reduced the formation of biofilm by Rm8530 (*p*-value of <0.0001). Treatment with ‘heat treated’ UD1022 CFS showed no significant difference compared to the control (*p*-value of 0.86).

UD1022 had no observable direct effects on Rm8530 growth; consequently, treatments of UD1022 culture filtrate supernatant (CFS) were tested for indirect influence on the Rm8530 functional phenotype of biofilm production. Biofilm formation by the symbiotic Rm8530 strain is required for efficient nodulation (González et al., 1996) and was evaluated by the semi-quantitative O’Toole assays (O’Toole et al., 2000) in treatments with UD1022 CFS. Biofilm of Rm8530 cultured with 5% by volume of UD1022 CFS was significantly reduced from that of control (Figure 2, *p* < 0.0001). Growth of Rm8530 with heat-treated CFS treatment resulted in restoration of control quantities of biofilm (Figure 2, *p* = 0.86), suggesting that the active factor of UD1022 CFS may be a heat-unstable molecule such as a protein.

### UD1022 Affects Rm8530 QS-Controlled Biofilm Gene Expression

**FIGURE 3:**
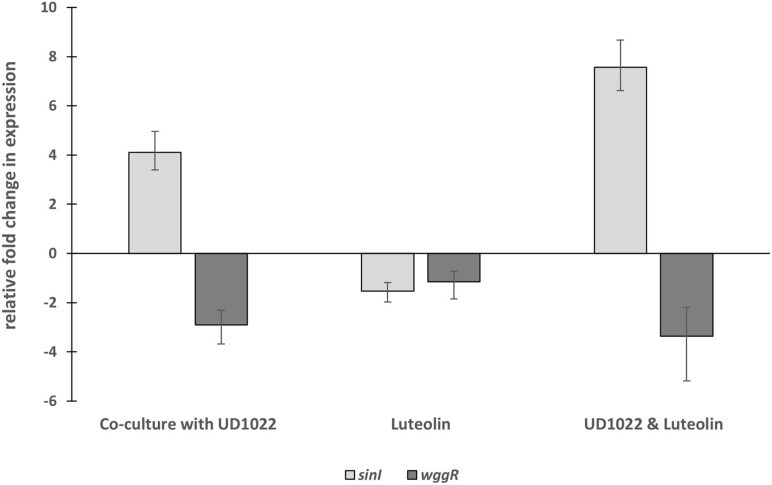
Relative fold changes in expression of Rm8530 *sinI* and *wggR* in co-culture with UD1022. Co-culture with UD1022 increased the relative expression of Rm8530 *sinI* by 4-fold. The presence of luteolin (which represents the condition of Rm8530 upregulating *nod* genes) doubled the effect of UD1022 on Rm8530 *sinI*, increasing expression to nearly 8-fold. Luteolin alone did not meet the threshold of 2-fold change in Rm8530 *sinI* expression. Co-culture with UD1022 decreased the relative expression of Rm8530 *wggR* by 3-fold. The presence of luteolin slightly enhanced the effect of UD1022 on Rm8530 *wggR*, increasing to 3.4-fold. Luteolin alone did not change Rm8530 *wggR* expression. Standard error bars reflect the range of the relative fold change in gene expression in response to the treatment.

**FIGURE 4:**
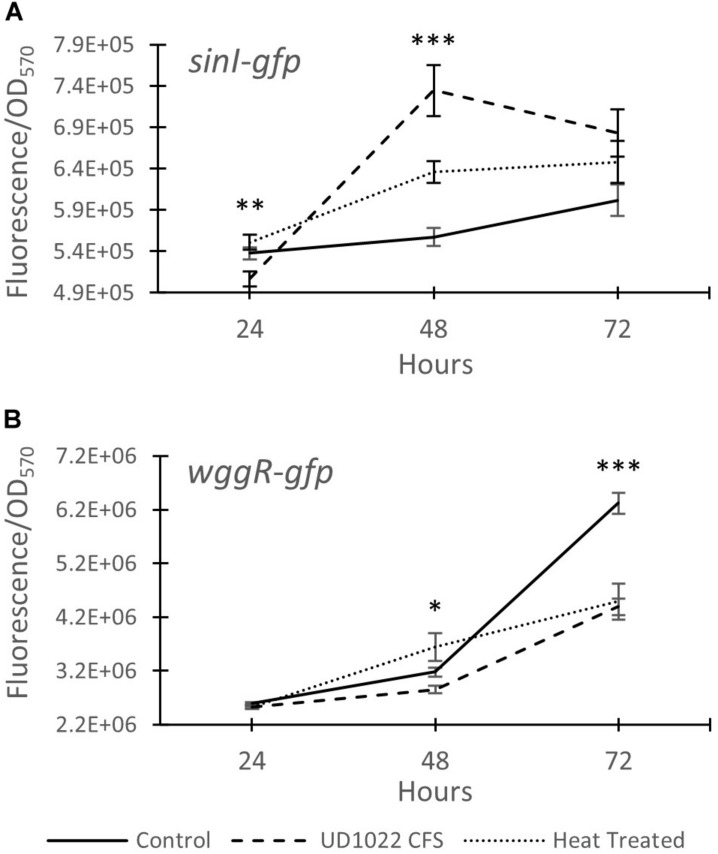
Expression of Rm8530 quorum sensing genes. **(A)** Average GFP activity (fluorescence/OD_570_) of the *sinI-gfp* fusion reporter. UD1022 treatment was significantly different from control and heat treatments at 24 hours. The differences between treatments at 48 hours is significant at *p*-value of <0.0001. **(B)** Average GFP activity of *wggR-gfp* fusion reporter. The differences between the treatments and the control at 48 hours is significant at *p*-value of 0.04, and 72 hours is significant with *p*-value of <0.0001. Averages for both assays are from 29 technical replicates and the bars at each time point present standard error. *indicates *p* < 0.05, **p < 0.005, and ***p < 0.0005.

The relative expression of two key Rm8530 QS genes were measured in response to co-culture with UD1022 CFS and in co-culture with live UD1022 cells using qRT-PCR. Rm8530 *sinI* relative gene expression increased by 4-fold and *wggR* relative gene expression decreased by nearly threefold in treatments grown with UD1022 (Figure 3). These UD1022 live-cell co-culture qRT-PCR results reflected the same trend of expression as observed in the GFP gene expression reporter assays treated with UD1022 CFS (Figure 4): upregulation of *sinI* and downregulation of *wggR*. Treatments with the *M. truncatula*-specific flavonoid luteolin (Peters et al., 1986) were included in the qRT-PCR expression analysis to evaluate possible plant host role in the interaction of the bacteria. Luteolin induces *nod* gene expression in *S. meliloti*, an important initial signaling mechanism to initiate legume–bacteria symbiosis. Rm8530 QS gene expression, as expected was not directly affected by the presence of luteolin alone. However, the presence of luteolin in Rm8530–UD1022 co-culture significantly enhanced the gene expression changes observed in bacteria co-cultures. The increase in *sinI* relative expression doubled to nearly 8-fold and *wggR* decreased expression was extended to 3.4-fold (Figure 3). This could indicate that, in the rhizosphere, plant signaling factors such as flavonoids may exacerbate the PGPR interactions causing the changes in QS gene expression.

### UD1022 Is Positive for QQ Activity Against Rm8530

The Rm8530 QS gene expression response patterns coupled with the restoration of WT Rm8530 biofilm formation in UD1022 heat treated CFS treatments suggest that UD1022 may be affecting Rm8530 QS through enzymatic activity of a protein. Interference of QS through interspecific enzymes is termed quorum quenching and can be enacted at several levels of QS regulation, including targeting signal biosynthesis, signal receptors, and direct cleavage of QS signal molecules, including AHLs (Fetzner, 2015). QQ enzymes have been characterized in many soil bacteria including *Agrobacterium* and *Bacillus* genera (Chan et al., 2016).

**FIGURE 5:**
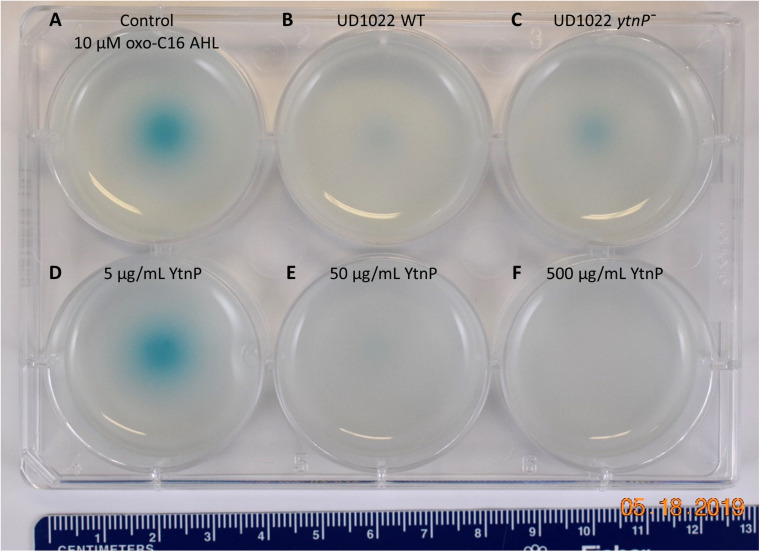
UD1022 quorum quenching biosensor assay plate. The biosensor KYC55-X-gal soft agar plate treated with UD1022-AHL co-cultures. From top left across **(A)** control treatments of standard AHLs with no UD1022. **(B)** QQ activity of UD1022 culture with 3-oxo-C16-AHL **(C)** UD1022 *ytnP*^–^ mutant cultured with AHL **(D)** 5 μg/mL pure UD1022 YtnP protein incubated with AHL, **(E)** 50 μg/mL YtnP protein, **(F)** 500 μg/mL YtnP protein. *Brightness of image increased by 20%, which did not increase pigment intensity or saturation.

QQ activity of UD1022 was assessed using the bioreporter strain *Agrobacterium tumefaciens* KYC55 to detect a wide range of AHLs and reports through β-galactosidase activity (Zhu et al., 2003). UD1022 cultures were incubated with 10 μM purified *N*-octanoyl-L-homoserine lactone (C8-AHL) or N-3-oxo-hexadecanoyl-L-homoserine lactone (3-oxo-C16-AHL) (Caymen Chemicals) for 24 h on KYC55 X-Gal plates. Treatments of UD122 with 3-oxo-C16-AHL showed significant reduction in detectable AHL signal as compared to AHL only (Figures 5A,B), while C8-AHL showed no discernable difference from the control (Supplementary Figure S2B). Thus, UD1022 displays QQ activity, which appears to be geared toward long-chain AHLs.

### UD1022 QQ Through the Lactonase YtnP Protein

Several classes of bacterial enzymes QQ through inactivating AHLs, including lactonases and acylases (Chan et al., 2016). A search of the literature for lactonases specifically identified in *B. subtilis* species yielded the putative lactonase YtnP protein in *B. subtilis* NCIB3610 (Schneider et al., 2012). The alignment of YtnP protein sequence with UD1022 returned a 98.44% identity. Using MUSCLE (Edgar, 2004) alignments of the reference protein *B. subtilis* strain 168 YtnP (NP_390867.1) and UD1022 YtnP sequences revealed the hallmark metallohydrolase HXHXDH and HXXGH metal binding motifs as well as a phosphorylated Ser36 residue. To determine if the UD1022 YtnP lactonase protein contributes to the QQ patterns observed, we introduced a *ytnP* deletion cassette in UD1022 (“UD1022 *ytnP*^–^”). In AHL co-incubation assays, UD1022 *ytnP*^–^ treatments with 3-oxo-C16-AHL showed that AHL degradation was less extensive than that of UD1022 WT (Figure 5C). It is likely that there are additional QQ active proteins produced by UD1022. Indeed, up to six other probable MBL-like fold sequences having the HXHXDH motif have been identified in UD1022 (data not shown).

The UD1022 specific *ytnP* sequence was submitted to the University of North Carolina School of Medicine Center for Structural Biology (NIH grant P30CA016086) for protein expression and purification. Purified YtnP protein was applied at three different concentrations to 10 μM concentrations of 3-oxo-C16-AHL and C8-AHL. The biosensor reporter showed no degradation of 3-oxo-C16-AHL with treatment of 5 μg/ml YtnP (Figure 5D). Long-chain AHL degradation comparable to UD1022 WT live cell treatments was observed with 50 μg/ml YtnP incubation (Figure 5E). Treatments of 500 μg/ml YtnP completely abolished detectable levels of 3-oxo C16-AHL (Figure 5F). Incubation of UD1022 YtnP with C8-AHL, interestingly, resulted in degradation of the short-chain AHL at 50 and 500 μg/ml (Supplementary Figures S2E,F, respectively). This demonstrates unequivocally that UD1022 QQ activity is carried out through the YtnP lactonase protein.

## Discussion

Understanding interactions of PGPR in consortia is critical for predicting rhizo-microbiome function in the environment and in agroecosystems. This is especially relevant as biological-based crop solutions become more widely marketed and adopted. Several examples of PGPR co-inoculations using *S. meliloti* resulting in significant improvements of *Medicago* spp. plant growth have been reported. Co-inoculation of *Delftia* spp. JD2, a diazotrophic, IAA-producing PGPR, with *S. meliloti* U143 onto *M. sativa* increased nodulation (Morel et al., 2011) and increased shoot and root dry weights by 13 and 34%, respectively (Morel et al., 2015). Fox et al. (2011) found dual inoculation of *S. meliloti* WSM419 and the PGPR *Pseudomonas fluroescens* WSM3457 onto *M. truncatula* enhanced nodule initiation rates, resulting in increased number of crown nodules and more overall N accumulation. *S. meliloti* B399, a commercial alfalfa inoculant closely related to strain Sm1021, co-inoculated with *Pseudomonas* spp. FM7d nearly doubled shoot dry weight and increased nodule number on *M. sativa* L. cv Bárbara SP (INTA Manfredi). Though co-inoculation of B399 with *Bacillus* spp. M7c had significantly higher shoot dry weight, it did not increase nodule number (Guiñazú et al., 2010).

However, not every instance of PGPR dual inoculation with *S. meliloti* has been reported to be beneficial. The dual inoculation of the PGPR *B. simplex* 30N-5 with *S. meliloti* 1021 onto *M. truncatula* resulted in no significant difference in shoot height, plant dry weight or nodule number over that of *S. meliloti* 1021 control (Maymon et al., 2015). This contrasted with their previous work, which showed beneficial growth effects of *B. simplex* 30N-5 when co-inoculated with *Rhizobium leguminosarum* bv. *viciae* 128C53 onto pea (*Pisum sativum*) (Schwartz et al., 2013). Our study using the *expR*+ *S. meliloti* strain Rm8530 co-inoculated with *B. subtilis* UD1022 also resulted in no significant enhancement of plant growth or nodule number. While other work has yet to query the mechanisms of bacterial interaction, which may account for the non-synergistic plant effects of these rhizobia-PGPR co-inoculations, this work reveals a potential, indirect mechanism of bacterial interaction.

Biofilm formation is important in soil and root-associated bacteria for motility and exchange of signals and metabolites (Angus and Hirsch, 2013; Bogino et al., 2013; Amaya-Gómez et al., 2015). *S. meliloti* biofilms have been shown to play a critical role in motility toward and initiation of nodulation with the *Medicago* spp. plant root (González et al., 1996; Pellock et al., 2000; Hoang et al., 2008). Here, we used Rm8530 biofilm formation as a functional reporter for negative activity by UD1022 and found clear evidence of UD1022 inhibition of Rm8530 biofilm formation. *S. meliloti* biofilm formation is dependent on an intact ExpR/SinI QS system, which is well described for both strains Rm1021 and its ExpR+ relative Rm8530. Importantly, the Rm8530 QS system has been shown to regulate a key symbiotically active component of their biofilms, the low-molecular-weight galactoglucans referred to as EPS II (Rinaudi and Gonzalez, 2009).

Based on the negative effect of UD1022 on Rm8530 biofilm, we hypothesized that UD1022 may be interfering with the QS-controlled molecular regulation of biofilm production. The Rm8530 QS genes *sinI* and *wggR* were selected to test the effect of UD1022 on the QS pathway, including upstream QS signal molecule synthesis (*sinI*) and downstream EPS II polymerization (*wggR*). Using UD1022 CFS treatments on Rm8530-*gfp* expression reporters and subsequent validation with qRT-PCR of live-cell co-cultures, we found that UD1022 significantly activated *sinI* transcription and reduced *wggR* transcription. McIntosh et al. (2009) described that *sinI*-promoter activation occurs at nearly 10-fold lower levels of AHLs than required for its downregulation. WggR activation requires the presence of the transcriptional regulator ExpR and the SinI-specific AHLs C_16:1_-AHL and oxo-C_16:1_-AHL (McIntosh et al., 2009; Gao et al., 2012). The *wggR*-*gpf* reporter in Rm8530 *sinI* background was more sensitive to C_16:1_-AHL than 3-oxo-C_16:1_-AHL. Expression of *wggR* increased in a dose-dependent manner with close to WT levels at 40–1500 nM C_16:1_-AHL and 200–1,500 nM oxo-C_16:1_-AHL (Gao et al., 2012). Consequently, UD1022 treatment appeared to mimic an expression pattern of the QS genes similar to their response to low AHL signal molecule concentration conditions.

**FIGURE 6:**
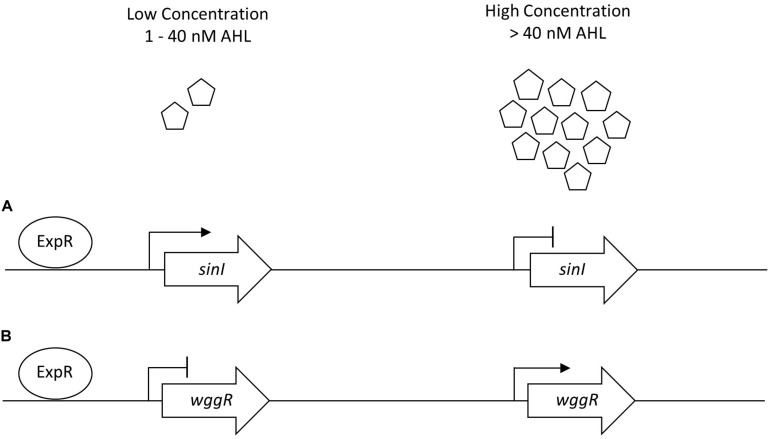
Generalized *sinI*/ExpR quorum sensing transcription model for *sinI* and *wggR*. Expression of genes are controlled on multiple levels including length of AHL chain and AHL concentration. **(A)** Low concentrations of AHL (1–40 nM) upregulate *sinI* expression in a positive feedback regulation. **(B)** Low concentrations of AHLs downregulate *wggR* expression. Adapted from Baumgardt et al. (2014).

The regulatory network of *S. meliloti* ExpR/SinI is intricately controlled through AHL acyl chain length, acyl chain substitutions, and concentration of AHL molecules (Bartels et al., 2007; Calatrava-Morales et al., 2018). Gene expression for *sinI* synthase is positively regulated by low concentrations of AHLs (1–40 nM) and negatively regulated by high concentrations of AHLs (>40 nM), allowing the ExpR transcriptional regulator of *sinI* to be sensitive to the AHL substrate it is responsible for producing (Baumgardt et al., 2014). The expression of *wggR* is oppositely regulated, requiring upwards of 150 nM C_16:1_ -AHL for increased *wggR* GFP expression reporter activity (Gao et al., 2012). Lower concentration of AHLs in UD1022 treatments would support the patterns of increased *sinI* and decreased *wggR* expression (schematic in Figure 6). QQ activities could be promising as a prospective tool to improve plant health and bypass antibiotic resistance in the development of biological products combating plant pathogens (Grandclément et al., 2016; Rodríguez et al., 2020). Many screening techniques have been utilized to identify QQ microbial isolates for this purpose (Tang et al., 2013; Last et al., 2016; Stein and Schikora, 2018). To better understand the capacity of UD1022 for QQ, we used the bioreporter strain *Agrobacterium tumefaciens* KYC55 in soft agar to detect both short- and long-chain AHLs. When co-cultures of UD1022 were incubated with 10 μM purified 3-oxo-C16-AHL for 24 h and applied to the bioreporter, expression of KYC55 β-galactosidase was greatly diminished. This reduction of detectable long-chain AHL demonstrates that UD1022 is capable of QQ activity. Response of the bioreporter to co-cultures of UD1022 with C8-HSL was no different from control treatments.

Many modes of QQ by bacteria have been identified with lactonase hydrolytic enzymes being highly described in *Bacillus* spp. (Kumar et al., 2015). The *B. subtilis* NCIB3610 putative lactonase YtnP protein sequence had high similarity to that found in UD1022 (Schneider et al., 2012). The YtnP lactonase is a metallolactamase and was found to target γ-butyrolactone of *Streptomyces griseus*. The UD1022 YtnP protein possesses the same hallmark metallohydrolase features of the NCIB3610 YtnP, including the HXHXDH motif, indicating that UD1022 YtnP is also a likely a QQ lactonase. The UD1022 *ytnP*^–^ mutant was employed in the AHL biosensor assay to test the role of the specific YtnP QQ activity. Rather than fully abolishing QQ, the partial degradation activity remaining may be due to redundant or multiple lactonase-like genes that continue to be expressed in the single *ytnP* mutant. The purified YtnP protein incubated with long- and short-chain AHLs showed clear and efficient QQ activity. Exogenous application of pure YtnP degraded C8-AHL, which was not observed in UD1022 live-cell assays. The possible substrate specificity of the YtnP lactonase protein may be more attributable to enzyme concentration rather than on acyl-chain length of the AHL. Lactonases characterized to date are described as having broad activity against a range of AHL acyl chain lengths and substitutions, though with variable active site affinities (Bergonzi et al., 2018).

QQ in the rhizosphere likely plays a large part in PGPR interactions and, consequently, in plant health outcomes. The presence of AHL molecules in the rhizosphere have been shown to directly elicit functional and beneficial responses from both non-legumes and legumes (Hartmann et al., 2014; Schikora et al., 2016; Hartmann and Rothballer, 2017). Several studies have employed the use of bacterial QQ lactonases to demonstrate the direct and indirect beneficial activities of AHLs and QS on plants. To identify novel QS-controlled proteins, an *S. meliloti* 1021 construct expressing the QQ lactonase AiiA was found to be significantly deficient in forming nodule initials within the first 12 h after inoculation (Gao et al., 2007). Zarkani et al. (2013) showed that *S. meliloti* producing 3-oxo-C14-AHL increased *Arabidopsis thaliana* resistance to *Pseudomonas syringe* pv *tomato* DC3000, while mutants heterologously expressing the *Agrobacterium tumefaciens* AttM lactonase did not.

**FIGURE 7:**
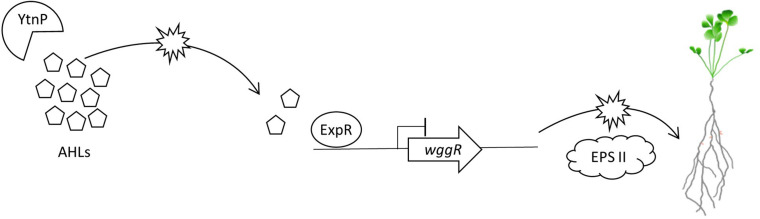
Model of proposed molecular QS and QQ interactions between *M. truncatula* PGPRs. *B. subtilis* UD1022 produces the lactonase YtnP which cleaves *S. meliloti* Rm8530 AHLs. Through quorum quenching, UD1022 YtnP reduces AHL concentrations, inhibiting the upregulation of symbiotically active EPS II genes. This may result in lower nodulation efficiency of Rm8530 in the presence of the PGPR UD1022.

The Rm8530 QS system is required to produce symbiotically active EPS II biofilms (Pellock et al., 2000, 2002; Hoang et al., 2004; Gurich and González, 2009). QS is also important in controlling bacterial cell population density, motility toward the plant root, and switching expression pathways from motility to nodulation (Bahlawane et al., 2008; Gao et al., 2012; Calatrava-Morales et al., 2018). The timing and coordination of these activities are intricately controlled through ExpR/SinI QS, and disruptions or interference through QQ has the potential to affect the efficiency and competency of these pathways. The lack of synergistic effects between Rm8530 and UD1022 may be explained, in part, through the QQ activity of UD1022 YtnP lactonase reducing Rm8530 AHL signal molecule concentration, leading to reduced expression of EPS II biosynthesis genes including *wggR*, and ultimately resulting in inhibition of efficient nodule initiation on *M. truncatula* roots (Figure 7).

## Conclusion

We show in our tri-trophic legume–symbiont–PGPR model system that the PGPR *B. subtilis* UD1022 does not synergistically increase *M. truncatula* plant growth or nodulation by the legume symbiont *S. meliloti* Rm8530. Though there is no direct growth inhibitory effect between the bacterial strains, indirect interactions contribute to the disruption of plant associative activities by the symbiont. UD1022 affects Rm8530 QS controlled biofilm formation through interference with the QS biosynthesis pathway. Further, UD1022 expresses the QQ lactonase YtnP, which cleaves the specific AHLs required to produce symbiotically active EPS II of Rm8530 biofilms. UD1022 likely delays or fails to promote Rm8530 nodulation through the QQ activity of lactonase YtnP and can inhibit synergistic plant growth promotion.

## Data Availability Statement

The raw data supporting the conclusions of this article will be made available by the authors, without undue reservation.

## Author Contributions

AR completed lab work pertaining to microbiology and genetic analysis. AR and HPB conceptualized the idea. PBB designed the experiments for the *Bacillus* mutants. All authors designed the experiments, edited, and contributed to the final manuscript, and approved for submission.

## Conflict of Interest

The authors declare that the research was conducted in the absence of any commercial or financial relationships that could be construed as a potential conflict of interest.

## Footnotes

1 https://www.genscript.com/ssl-bin/app/primer

2 www.appliedbiosystems.com

3 www.eppendorf.com
