# Supplementary material for: Quorum Quenching Activity of the PGPR Bacillus subtilis UD1022 Alters Nodulation Efficiency of Sinorhizobium meliloti on Medicago truncatula
Source: Front Microbiol. 2021 Jan 15;11:596299. doi: 10.3389/fmicb.2020.596299 (PMC7843924; doi:10.3389/fmicb.2020.596299)
Supplement: Supplementary file 1 [file Table_1.DOCX]

Supplementary Material

**Supplementary Figures**


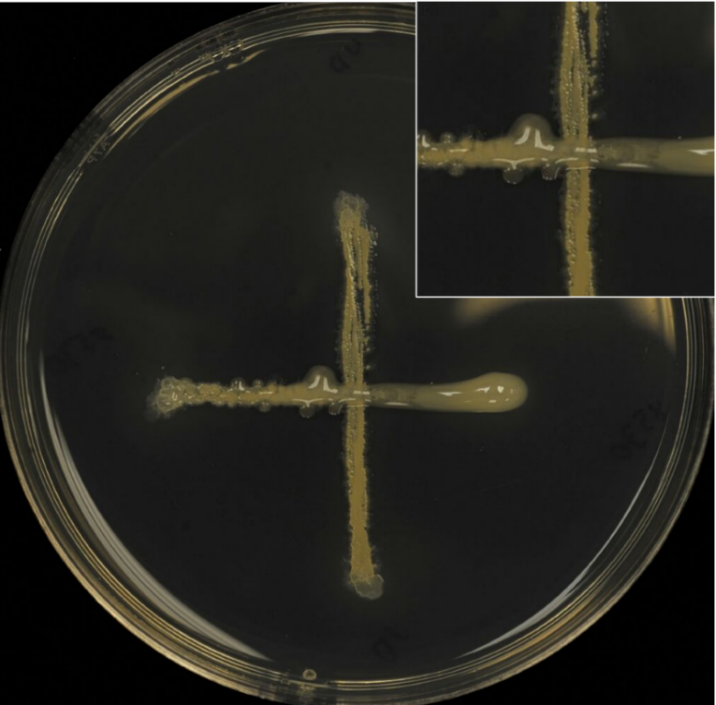


**Supplementary Figure 1.** Cross-streak assay. The bacteria *S. meliloti* strain Rm8530 (horizontal) and *B. subtilis* strain UD1022 (vertical), were streaked together to test for growth inhibition. The two bacteria demonstrated no growth inhibition on solid or in liquid media (data not shown).


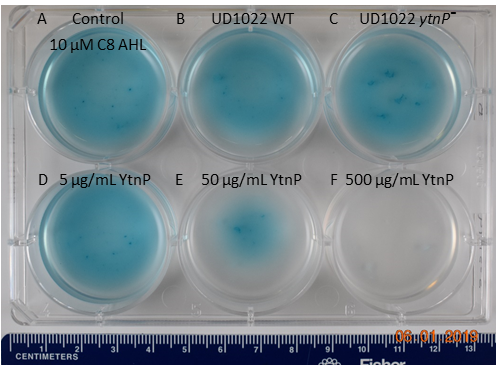


**Supplementary Figure 2.** UD1022 Quorum quenching biosensor assay plate. The biosensor KYC55-X-gal soft agar plate treated with UD1022-AHL co-cultures. From top left across: **(A)** control treatments of standard AHLs with no UD1022. **(B)** QQ activity of UD1022 culture with C8-HSL **(C)** UD1022 *ytnP*¯ mutant cultured with AHL **(D)** 5 µg/mL pure UD1022 YtnP protein incubated with AHL, **(E)** 50 µg/mL YtnP protein, **(F)** 500 µg/mL YtnP protein.
